# Protocol of a nation-wide post-bereavement survey on quality of hospice and palliative care: J-HOPE 5 study

**DOI:** 10.1186/s12904-024-01600-6

**Published:** 2024-12-03

**Authors:** Maho Aoyama, Masanori Mori, Tatsuya Morita, Satoru Tsuneto, Mitsunori Miyashita

**Affiliations:** 1 Department of Palliative Nursing, Health Sciences, Tohoku University Graduate School of Medicine, 2-1 Seiryo-machi, Aoba-ku, Sendai, 980-8575 Miyagi Japan; 2 Department of Palliative and Supportive Care, Palliative Care Team, Seirei Mikatahara General Hospital, 3453 Mikatahara-cho, Chuo-ku, Hamamtsu, 433-8558 Shizuoka Japan; 3 Department of Human Health Sciences, Kyoto University Graduate School of Medicine, 54 Kawaharacho, Shogoin, Saikyo-ku, Kyoto, 606-8507 Japan; 4 Department of Nursing, Yamagata Prefectural University of Health Sciences, 260 Kamiyanagi, Yamagata, 990-2212 Yamagata, Japan

**Keywords:** Palliative care, Bereavement, Evaluation, J-HOPE study, Japan

## Abstract

**Background:**

Maintaining quality of care and improving the quality of life (QOL) of patients and their families are important issues in palliative care. Therefore, there is a need to continuously evaluate the quality and outcomes of the care provided. In Japan, the Japan hospice and palliative evaluation (J-HOPE) study series has been conducted every three to four years since 2010, and we will conduct the fifth J-HOPE study (J-HOPE5). In the present paper, we describe the protocol of the J-HOPE5 study. The main objectives are: (1) to evaluate the processes, structures and outcomes of care at hospices or palliative care units; (2) to examine bereaved family members’ self-reported psychosocial condition, such as grief and depression as bereavement outcomes; (3) to provide data to ensure and improve the quality of care provided by participating institutions via feedback based on the results from each institution; and (4) to provide clinical and academic information regarding the implications of various issues in palliative care by conducting specific research.

**Methods:**

We will conduct a cross-sectional, anonymous, self-reported questionnaire survey. In total, 153 institutions have agreed to participate in this study, meaning that approximately 12,240 bereaved family members (*n* = 80/institution) will be sent a questionnaire.

**Discussion:**

This is one of the largest cross-sectional bereavement surveys to evaluate the quality of specialized palliative care for patients with cancer, both in Japan and worldwide. The large sample size of this study will enable wide analyses of specific targets and topics.

## Background

Maintaining quality of care and improving the quality of life (QOL) of cancer patients and their families are important issues in palliative care. Therefore, there is a need to continuously evaluate the quality and outcomes of the care provided. Direct evaluation by the patients themselves is considered the most reliable method for evaluating care. However, especially in the case of palliative care, it is often methodologically difficult due to the patient’s poor general condition, impaired consciousness, and cognitive impairment. For this reason, evaluation of the quality of hospice and palliative care based on reports from family members and bereaved families has become the standard method of evaluation worldwide [1, 2].

The history of nationwide surveys on quality assessment of specialized palliative care in Japan has been previously published [3–7], and continuous efforts in this regard have been made for more than two decades. In line with this, several scales have been developed to assess the three aspects of care proposed by Donabedian [8]: structure, processes and outcomes, such as the Good Death Inventory (GDI) [9] and the Care Evaluation Scale (CES) [10, 11]. In response to these scales, the Japan Hospice and Palliative Care Evaluation (J-HOPE) study (J-HOPE study) was conducted from 2007 to 2008 to evaluate the quality of palliative care for cancer patients in Japan [6]. This was the first large-scale national survey of general hospitals, hospice and palliative care units, and clinics, etc., in Japan, and used CES, GDI, and other evaluation scales to assess the quality of end-of-life care from multiple perspectives, consistent with the Donabedian model [8, 12]. Three years later, in 2010, the J-HOPE2 study was conducted, and at four year intervals thereafter, the J-HOPE3 [4] and J-HOPE4 [3] studies were conducted in 2014 and 2018, respectively. These study series by our country have been highly evaluated worldwide.

We will conduct the fifth J-HOPE (J-HOPE5) study. Originally, the study was scheduled to be conducted in 2022, but its postponement was unavoidable due to the significant impact of the COVID-19 pandemic on the palliative care delivery system, including closure of palliative care wards and downsizing and restriction of patient visits. The four previous J-HOPE studies have contributed to quality assurance and improvement of hospice and palliative care by identifying changes in the quality of hospice and palliative care over time, along with areas for improvement, feeding the results back to the participating facilities, and publicizing the study results domestically and internationally. Since it is difficult to obtain proxy evaluations of bereaved families or cooperation from facilities in general wards and home clinics, where visitation restrictions and access to the services are still largely in place, only palliative care wards are targeted in this study. While the impact of the pandemic cannot yet be said to be completely over, it is important to continue to assess the quality of care at fixed intervals, and it is expected that additional information will be gleaned under this extraordinal situation after/with COVID-19.

As in past J-HOPE studies, the main objectives of the J-HOPE5 study are the following: (1) to evaluate the processes, structures and outcomes of care at hospices and palliative care units; (2) to examine bereaved family members’ self-reported psychosocial condition, such as grief and depression, as bereavement outcomes; (3) to provide data to ensure and improve the quality of care provided by participating institutions via feedback based on the results from each institution; and (4) to provide clinical and academic information regarding the implications of various issues in palliative care by conducting specific research.

## Methods

We will conduct an anonymous, cross-sectional, self-reported questionnaire survey between May and June 2024. A document explaining the J-HOPE4 study’s aims and procedures will be included along with the questionnaire, and the return of a completed questionnaire will be considered as consent to participate in the study. A ballpoint pen will be included in the envelope as an incentive to participate. Participants will be asked to return the completed questionnaire to the secretariat office (Tohoku University) within 2 weeks. We will send a reminder to non-responders a month after sending the questionnaire. If they do not wish to participate in the study, they will be asked to check a “no participation” box and return the incomplete questionnaire. Ethical approval for the study has been granted by the institutional review board of Tohoku University (ID: 2023-1-872), and we will obtain approval to conduct the study from all participating institutions.

### Participants

As of December 2022, research requests were sent to the medical personnel in charge of the palliative care wards of 460 facilities that are open to the public at the respective prefectural/regional bureaus of Health and Welfare, and, as of March 2024, 153 facilities have agreed to participate. To identify potential subjects, we will ask each institution to identify and list up to 80 bereaved family members of patients who died prior to January 31st, 2024. However, deaths prior to January 31, 2022 will not be included. If the number of deaths meeting the eligibility criteria during the period is 80 or fewer, all cases will be included. The inclusion criteria are as follows: (1) death due to cancer, (2) the patient was aged 20 years (the age at which one is considered an adult in Japan) or older at the time of death, and (3) the bereaved family member is aged 20 years or older. The exclusion criteria are as follows: (1) the patient received palliative care for less than 3 days; (2) the bereaved family member is unavailable or cannot be identified; (3) death was associated with treatment or occurred in an intensive care unit; (4) the potential participant suffered serious psychological distress, as determined by the primary physician and a nurse; and (5) the potential participant is incapable of completing the self-reported questionnaire because of health issues, such as cognitive impairment or visual disability.

### Questionnaire

The questionnaire consists of common items and specific additional questions, and the maximum length of the questionnaire sent to individual patients is about 12 pages long. Common questionnaires will be sent to all participants, while specific research questionnaires will be randomly inserted into the documents sent to the participants. The topics of the specific research are described in Table 1. Common questionnaires include the following scales/questions, as well as the participants sociodemographic data (i.e. age, sex, relationship with the deceased, educational background and annual income).

**Table 1:** List of specific studies

Specific topics related to palliative care/ end-of-life care
1. Association between “attachment styles and continued bonding with the deceased” and “bereavement-related depression and prolonged grief syndrome”
2. Regrets of families of terminally ill cancer patients
3. A study on family evaluation of bereavement care at the time of death
4. Bereaved families’ experiences and thoughts on eye donation/organ donation
5. Advantages of online visitation, disadvantages and points to be improved in end-of-life
6. Music that encouraged the bereaved when they were depressed
7. Support needed by families of cancer patients and the medical resources available
8. Unfinished business of a family member who has lost a patient in a palliative care unit
9. Desirable care for family members who have expressed feelings of suicide ideation
10. Survey on the perception that draining ascites weakens patients
11. Experiences of seasonal events and special occasions for terminally ill cancer patients
12. Perceptions of signs of breathing in the perimortem period: is the process of death acceptable for the families?
13. Family relationships and family conflicts related to caregiver affirmation
14. Preparedness of families of cancer patients for bereavement
15. Emotional burden on family members in terms of phone calls from the hospital
16. Desirable care and family experience of bridging the gap between the patient and family in communicating thoughts and feelings at the end of life
17. Health literacy of bereaved family members who experienced caring for a cancer patient in relation to the quality of palliative care and the mental state of the bereaved family members.
18. Current status and need for psychological support for bereaved families of cancer patients
19. Research on the use of peer support by bereaved family members of cancer patients
20. Research on the timing of life expectancy notification after completion of anticancer treatment
21. Loneliness in families with cancer patients experiencing bereavement
22. Discussion of prognostic and functional predictors of prognosis and quality of death
23. Distress associated with the hospitalization of terminal cancer patients: Family member’s experience
24. Preferred care for family members while waiting for admission to a palliative care unit
25. Opinion of bereaved family members regarding the interview for admission to the palliative care ward
26. Self-stigma of cancer patients from the survivor’s perspective

### Care evaluation scale (CES) short version

We will use the revised short version of the CES [11] to evaluate the structure and processes of end-of-life care. The original version of the CES includes 10 domains and 28 attributes. The response options are in the form of a 6-point Likert scale (6: highly agree; 5: agree; 4: somewhat agree; 3: somewhat disagree; 2: disagree; 1: highly disagree). Total scores will be transferred to a 100-point scale, with higher scores indicating better care. The short version of the CES consists of 10 representative items from each domain, and the validity and reliability of the scale have been previously confirmed.

### Good death inventory-short version

We will use the short version of the GDI [9] to measure patients’ attainment of a good death from the perspective of bereaved family members. The original version of the GDI consists of 10 core and 8 optional domains and 54 attributes. The 10 core domains evaluate the attributes that Japanese people consistently rate as important, and the eight optional domains evaluate attributes that are rated as important, albeit inconsistently, and depend upon individual values. The short version of the GDI consists of 18 representative items from each domain, and the validity and reliability of the scale have been confirmed. Participants will evaluate each attribute using a seven-point Likert scale (1: absolutely disagree, 2: disagree, 3: somewhat disagree, 4: unsure, 5: somewhat agree, 6: agree, and 7: absolutely agree). The total score will be calculated by summing the scores for all attributes, with a high total score indicating the attainment of a good death.

### Brief grief questionnaire

We will use the Brief Grief Questionnaire (BGQ) to assess complicated grief (CG). The BGQ was developed by Shear et al. [13], and the reliability and validity of the Japanese version have been confirmed [14]. Although the BGQ was originally developed to assess CG in people who had lost a loved one in the September 2011 attacks, since Fujisawa et al. used the questionnaire in the general Japanese population, including bereaved individuals who had lost a loved one to cancer [15], the previous J-HOPE studies have adopted this scale to assess CG in bereaved family members of patients with cancer [3, 4, 16]. A total score of 8 or higher indicates that the respondent is likely to develop CG, scores of 5–7 indicate subthreshold CG, and scores of < 5 indicate that the respondent is unlikely to develop CG.

### Patient health questionnaire 9

We will use the Patient Health Questionnaire 9 to assess depressive symptoms. This is a widely accepted instrument that consists of nine items to assess depressive symptoms, used as a brief diagnostic tool, and measures the severity of depression in both clinical practice and research; the reliability and validity of the scale have been previously confirmed [17, 18]. Each of the nine items concerns the extent to which a particular depressive symptom has bothered the respondent in the preceding 2 weeks. Responses are provided on a scale ranging from 0 (not at all) to 3 (nearly every day), and total scores range from 0 to 27. Scores of 5, 10, 15 and 20 represent valid cutoff points indicating minimal, mild, moderate, moderately severe and severe depression, respectively.

### Restrictions on visiting the patient due to COVID-19

This study targets family members who experienced bereavement from January 2022 to January 2024, a period during which visits to patients in Japan were greatly restricted by COVID-19. Since the degree of restriction varied with time and facility, we included the following original questions regarding visitation restrictions due to COVID-19: whether the number of people who could visit and the amount of time allowed per visit were limited; impact of restricted visitation (degree of hardship on the family and patient, degree of anxiety due to not knowing the patient’s condition); response of the health care provider to visitation restrictions (availability to talk remotely by phone or web, explanation of the patient’s condition by the health care provider).

### Characteristics of participating institutions

We will ask participating institutions to describe the treatment available, the bereavement care offered for family members, and the structure of the patient care provided. The structure of care at each institution includes items such as the details of religious affiliations and the numbers of medical staff members, beds, rooms and patients. Items concerning available treatments, such as surgery under general anesthesia, intravenous or oral chemotherapy, intravenous hydration, intravenous hyperalimentation, pleuro- and abdominocentesis, nerve block, physiotherapy/rehabilitation, and other complementary and alternative medicines, will be evaluated. In addition, molecular targeted therapy, hormone therapy, radiation therapy, red-blood cell transfusion, platelet transfusion, and complementary and alternative medicines, such as Maruyama and peptide vaccine hypodermic injections, thermotherapy, aromatherapy, reflexology, music therapy, lymphedema therapy by certificated specialists, and referral to available specialists, will also be evaluated.

### Data analysis

Based on the number of participating facilities (*n* = 153) currently planned and the number of listings from each facility (*n* = 80), we estimate that the total number of eligible participants will be approximately 12,240. Based on previous J-HOPE studies, we expect a response rate of 60%. Hence, the number of responses eligible for analysis is expected to be 7,344.

With regard to the main objectives, we will calculate the mean values of the evaluation scales of structure, processes and outcomes of care from the survivor’s perspective separately for each facility, and calculate their distribution overall and at each facility. Univariate and multivariate analyses will be conducted using these as objective variables and the survivor and facility backgrounds as explanatory variables, in order to clarify the factors that cause differences between facilities. In addition, the distribution of GDI, CES, PHQ-9 and BGQ and their related factors will be clarified.

## Discussion

This paper outlines the study protocol of the J-HOPE5 study. This will be the fifth study in the J-HOPE study series, which is one of the largest cross-sectional surveys in Japan and worldwide, for evaluating the quality of end-of-life care. This study has several strengths and limitations as follows:

### Strengths

One of the strengths of this study is the large anticipated sample size. Therefore, the findings are expected to be generalizable to other settings. Due to their large sample size, previous J-HOPE studies have allowed for analyses of issues and targets (e.g., rare cancers and specific topics) for which it would be difficult to separately recruit subjects [19, 20]. Second, this study includes many specific studies that are expected to be useful for clinical practice.

### Limitations

First, the participants would be bereaved family members of patients with cancer who died in hospices or palliative care units. Therefore, the results might be limited to patients and families who receive specialized palliative care. Second, the number of institutions planning to participate in this study is 153, which constitutes approximately 33% of the registered hospice or inpatient palliative care units in Japan. In addition, there might be recall bias because of the retrospective nature of the study. However, according to some studies, considering both recall bias and the grieving process, 3–12 months after death might be an appropriate time frame for participant inclusion [21, 22]. Third, in terms of questions asking about the deceased patient’s condition/quality of care at the end of life (i.e. CES and GDI), the assessment could be limited due to visitation restrictions due to COVID-19. However, the ability to evaluate the quality of care in such a context and to compare it with previous data might be a strength of this study.

## Data Availability

No datasets were generated or analysed during the current study.

## Abbreviations

J-HOPE study: The Japan hospice and palliative care evaluation study
GDI: Good death inventory
CES: Care evaluation scale
BGQ: Brief grief questionnaire
PHQ-9: Patient health questionnaire 9
